# Harnessing promoter elements to enhance gene editing in plants: perspectives and advances

**DOI:** 10.1111/pbi.14533

**Published:** 2025-02-27

**Authors:** Nikita Gondalia, Luis Felipe Quiroz, Linyi Lai, Avinash Kumar Singh, Moman Khan, Galina Brychkova, Peter C. McKeown, Manash Chatterjee, Charles Spillane

**Affiliations:** ^1^ Agriculture, Food Systems and Bioeconomy Research Centre, Ryan Institute University of Galway Galway Ireland; ^2^ Viridian Seeds Ltd. Cambridge UK

**Keywords:** Promoters, plant gene editing, CRISPR/Cas, synthetic promoters, promoter editing, synthetic biology

## Abstract

Genome‐edited plants, endowed with climate‐smart traits, have been promoted as tools for strengthening resilience against climate change. Successful plant gene editing (GE) requires precise regulation of the GE machinery, a process controlled by the promoters, which drives its transcription through interactions with transcription factors (TFs) and RNA polymerase. While constitutive promoters are extensively used in GE constructs, their limitations highlight the need for alternative approaches. This review emphasizes the promise of tissue/organ specific as well as inducible promoters, which enable targeted GE in a spatiotemporal manner with no effects on other tissues. Advances in synthetic biology have paved the way for the creation of synthetic promoters, offering refined control over gene expression and augmenting the potential of plant GE. The integration of these novel promoters with synthetic systems presents significant opportunities for precise and conditional genome editing. Moreover, the advent of bioinformatic tools and artificial intelligence is revolutionizing the characterization of regulatory elements, enhancing our understanding of their roles in plants. Thus, this review provides novel insights into the strategic use of promoters and promoter editing to enhance the precision, efficiency and specificity of plant GE, setting the stage for innovative crop improvement strategies.

## Introduction

Clustered regularly interspaced short palindromic repeats (CRISPR)/Cas‐based plant gene editing (GE) systems have garnered significant attention in recent years due to their remarkable precision, programmability and versatility (Yin *et al*., 2017) compared with other genome editing systems, such as zinc‐finger nucleases (Zhang *et al*., 2010), transcription activator‐like nuclease (TALENS) (Zhang *et al*., 2013). In these systems, the effectiveness of nuclease expression relies significantly on the strategic utilization and precise selection of promoters for GE (Villao‐Uzho *et al*., 2023). Positioned upstream of the 5′UTR region, promoters play a central role in recruiting the transcription machinery and regulating gene expression through their interactions with transcription factors (TFs) and RNA polymerases (Archuleta *et al*., 2024; Thomas and Chiang, 2006). In most eukaryotes, three main types of RNA polymerases are found. RNA polymerase I (RNA Pol I) is responsible for synthesizing the precursor of ribosomal RNA (rRNA), while RNA polymerase II (RNA Pol II) is involved in synthesis of mRNA precursors, and RNA polymerase III (RNA Pol III) is responsible for producing the precursors of transfer RNAs (tRNAs), 5S ribosomal RNAs and other miRNAs (Hummel and Liu, 2022; Yang *et al*., 2023). Additionally, in plants, RNA polymerases IV and V play roles in synthesizing miRNAs associated with gene silencing (Haag and Pikaard, 2011; Xie *et al*., 2023). Among these, RNA Pol II promoters transcribe all protein‐coding genes and many noncoding RNAs, whereas RNA Pol III promoters exhibit high and steady transcriptional activity, accounting for approximately 40% of total RNA (Kor *et al*., 2022; Schier and Taatjes, 2020).

**Figure 1:**
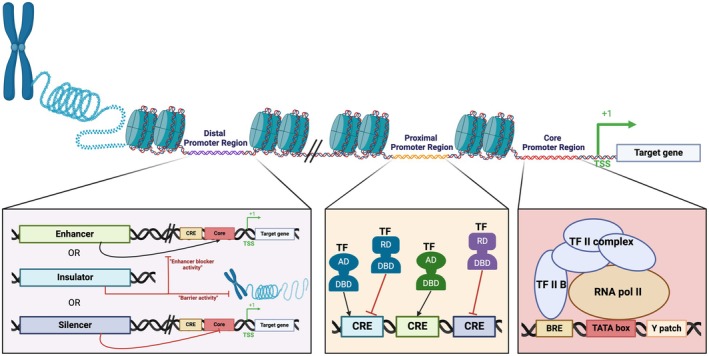
Schematic representation of basic components in a promoter. Core promoter region of the plant is located near to transcription start site (TSS), containing TATA box, Y patch and B recognition element (BRE), where RNA Polymerase II (RNA Pol II) and transcription factors (TF II complex) bind. The proximal promoter region situated near the core region, contains Cis‐regulatory elements (CREs). The distal promoter region is located several hundred (or even thousand) base pairs away from the TSS and comprises enhancers, insulators and silencers. AD: activation domain; RD: repression domain; DBD: DNA binding domain. Created with BioRender.com.

RNA Pol II promoters are categorized into constitutive, inducible, tissue/organ‐specific and synthetic types, whereas RNA Pol III works with promoters with different architectures, the U3 and U6 promoters (Kor *et al*., 2022; Potenza *et al*., 2004). Promoter elements associated with Pol II regulate the spatiotemporal expression of genes and consist of core, proximal and distal regions (Dutt *et al*., 2014). The core promoter, spanning approximately 50 bp upstream of the transcription start site (TSS), is critical for the binding of RNA Pol II and general transcription factors (GTFs), facilitating the formation of the pre‐initiation complex (PIC) (Andersson and Sandelin, 2020; Haberle and Stark, 2018). Key features in most eukaryotes include the TATA box, initiator (Inr) and B recognition element (BRE). The TATA box, characterized by the sequence TATA(A/T)A(A/T), is recognized by the TATA‐binding protein (TBP), essential for the recruitment of the basal transcription machinery (Smale and Baltimore, 1989; Smale and Kadonaga, 2003). In plants, the short motifs made up of pyrimidine bases known as the TC motif, or Y patch, have been identified as a potential core promoter element (Figure 1) (Bernard *et al*., 2010). Proximal and distal promoter regions may contain other regulatory elements such as enhancers, insulators and silencers (Andersson and Sandelin, 2020). The proximal promoter is typically enriched with transcription factor binding sites (TFBS), forming conserved clusters known as cis‐regulatory elements (CREs) that regulate gene expression. Distal elements, located further from the TSS, can also play significant roles; enhancers facilitate RNA Pol II recruitment, while insulators prevent unwanted interactions between enhancers and promoters (Biłas *et al*., 2016; Hernandez‐Garcia and Finer, 2014; Vanaja and Yella, 2022). Recent findings indicate that enhancers in plants are position‐dependent, contrasting with the position‐independent nature seen in animals and yeast, highlighting a key difference in transcriptional regulation (Voichek *et al*., 2024). Insulators possess ‘enhancer blocker’ and ‘barrier’ activities to fine‐tune transcriptional control (Brasset and Vaury, 2005; Melnikova *et al*., 2020). Silencers, when bound by TFs, actively repress target gene expression, contributing to precise, tissue‐specific expression patterns (Figure 1) (Ogbourne and Antalis, 1998; Schmitz *et al*., 2021). In plants, gene expression is primarily regulated at the transcriptional level. The accessibility of CREs, along with their interaction with trans‐acting TFs and epigenetic regulators (ERs), dictates spatiotemporal expression. This interplay among TFs, RNA Pol II, GTFs and ERs orchestrates gene expression in a condition‐dependent manner (Kaufmann *et al*., 2010; Yaschenko *et al*., 2022).

In the CRISPR/Cas system, the expression of Cas9 and other Cas proteins like CRISPR‐Cpf1 have usually been driven by strong Pol II promoters. Cpf1 is shorter than SpCas9, recognizes thymidine‐rich PAM sites and does not require trans‐acting crRNA (tracrRNA), making it ideal for multiplex genome editing systems (Li *et al*., 2018; Tang *et al*., 2017). Moreover, the recent discovery of transposon‐associated nuclease proteins, TnpBs, encoded by Cas12a's ancestral gene, which function as RNA‐guided nucleases, offers new opportunities for genome editing (Xiang *et al*., 2024). TnpBs are much smaller than Cas9 and Cas12a, making them ideal for developing GE systems using viral vectors. Recently, hypercompact GE systems, such as ISDra2 from *Deinococcus radiodurans*, have been found to increase editing efficiency (Karmakar *et al*., 2024). Furthermore, a miniature plant genome editor system has been successfully developed using the smaller IsDge10 nuclease, a TnpB protein from *D. geothermalis*, driven by strong constitutive promoters (Zhang *et al*., 2024b). Apart from Cas9 and Cas12a, Cas12b (Ming *et al*., 2020; Wang *et al*., 2020a), Cas12f1 (Bigelyte *et al*., 2021) and the hypercompact Cas12j2 GE system, driven by strong constitutive promoters (Liu *et al*., 2022a), have all been utilized for plant GE. In addition to Cas proteins and their variants, an alternative and precise GE tool, the Cas‐CLOVER, which is a novel high‐fidelity site‐specific nuclease system, also shows high precision. This system uses two guide RNAs (gRNAs), which direct the Clo051 endonucleases. Cas‐CLOVER is a fusion protein of a ‘dead’ Cas9 (dCas9) with Clo051 endonucleases, creating double‐strand breaks only when both gRNAs target the correct site (Madison *et al*., 2022). In plant GE by CRISPR/Cas technology, the expression of Cas9 is primarily driven by RNA Pol II promoters, while sgRNA is mainly expressed under the control of RNA Pol III promoters (U3 and U6) (Kor *et al*., 2022; Tang *et al*., 2016). However, there are some instances in which single or polycistronic sgRNAs are expressed by RNA Pol II promoters (An *et al*., 2021; Čermák *et al*., 2017; Zhang *et al*., 2021, 2024b; Zheng *et al*., 2023).

The constitutive Pol II promoters have proven to be highly effective in plants and have been extensively utilized in plant GE over the past two decades in various crops. Among these, the viral‐derived promoter 35S from *Cauliflower Mosaic* virus (*pCaMV35s*), and plant‐derived *Ubiquitin* promoters from different species are the most widely used, which underscores the effectiveness of constitutive promoters for plant GE regardless of their species of origin (Lee *et al*., 2023a; Sun *et al*., 2015; Villette *et al*., 2024; Wu *et al*., 2024). Moreover, the utilization of endogenous Pol II and Pol III promoters to drive the expression of Cas and sgRNA has proven to be more effective for GE by CRISPR/Cas (Di *et al*., 2019; Long *et al*., 2018; Massel *et al*., 2022; Ren *et al*., 2021a). In addition to GE facilitated by constitutive promoters, tissue‐specific genome editing (TSGE) has emerged as a highly efficient technology for achieving precise genetic modifications in plants. TSGE relies on the utilization of tissue, organ or cell‐specific promoters (TSPs) to direct the expression. The incorporation of TSPs in genome editing by CRISPR/Cas in plants can facilitate the preferential expression of sgRNA/Cas9 complex within specific cell types or under inducible conditions, allowing precise spatiotemporal regulation of target gene expression with minimum potential lethal mutations (Decaestecker *et al*., 2019; Singha *et al*., 2021). Furthermore, with the advancement of high‐throughput synthetic biology techniques and the integration of artificial intelligence, the manipulation or construction of artificial biological components has become achievable. This capability facilitates the modification or synthesis of CREs, which can recruit a tissue‐specific or inducible transcriptional activator. Consequently, synthetic promoters can be created, providing precise control over gene expression in desired tissues or under specific conditions (Huang *et al*., 2021; Yaschenko *et al*., 2022). These promoters offer a solution to the limitations inherent in native promoters, including constraints related to transcription efficiency, strength and induction conditions, which show potential applications in precise GE and crop breeding (Wurtzel *et al*., 2019; Yasmeen *et al*., 2023). While polymorphisms in coding regions can impact gene function leading to desirable traits, natural or induced mutations in promoter regions can also lead to new or improved biological functions and desirable traits. Promoter modifications can open new opportunities for targeting non‐coding regions through GE in plants, with the potential for trait improvements. This review provides novel insights on the utilization of various promoters for plant transgene expression, aiming to revolutionize plant GE for sustainable development. The objective is to provide direction for the selection of an appropriate promoter and promoter region editing for plant GE without adverse effects on non‐transgenes.

## Harnessing constitutive pol II promoters for plant GE

**Figure 2:**
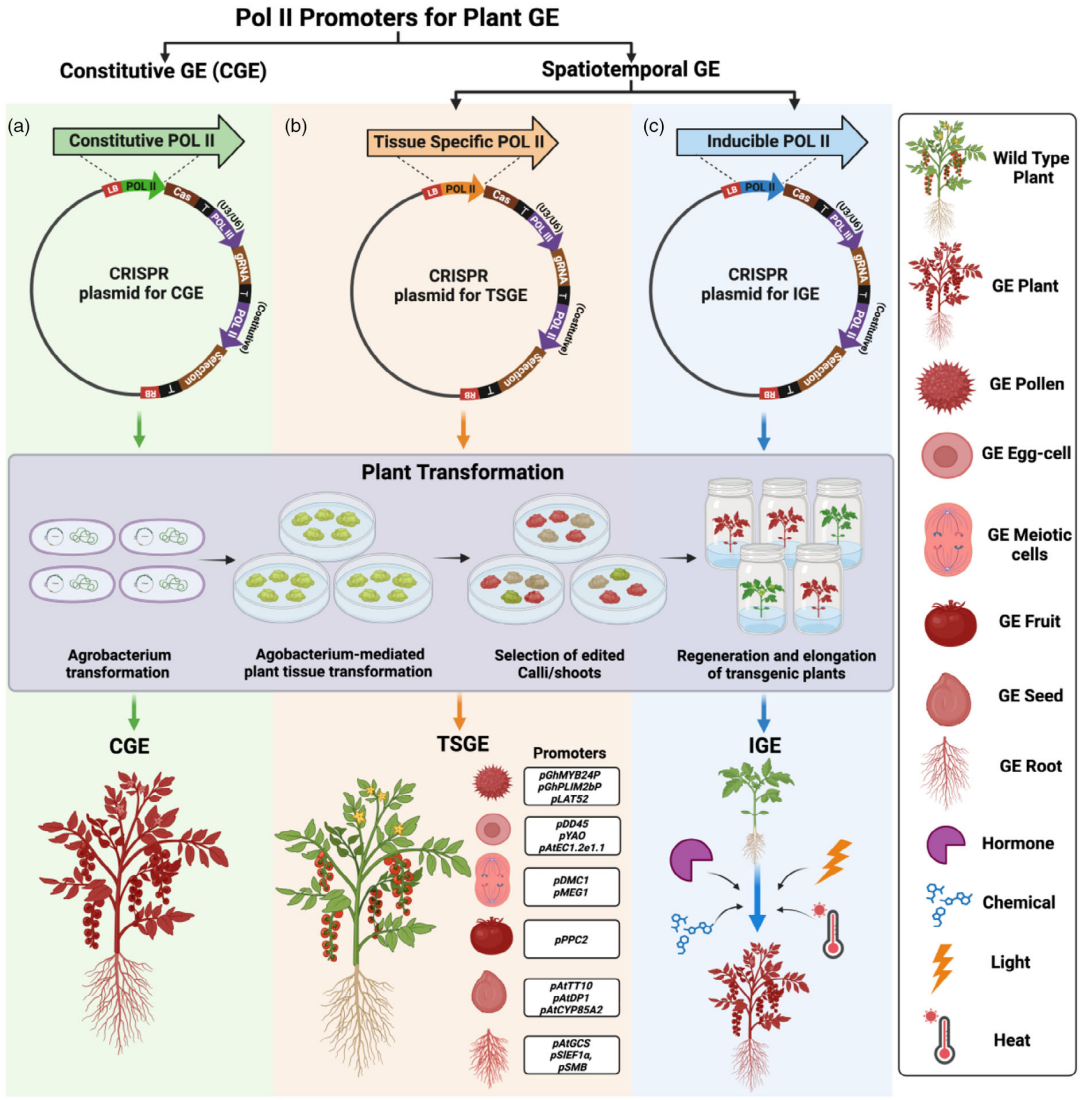
Promoters utilized for plant GE through CRISPR/Cas system. (a) Constitutive plant GE (CGE) thought constitutive Pol II promoter‐driven Cas9. (b) Tissue‐specific plant GE (TSGE) utilizing tissue/organ/cell‐specific promoter‐driven Cas expression. (c) Inducible plant GE (IGE) by harnessing inducible promoter. The box in the middle depicts the steps of plant transformation with *Agrobacterium* containing CRISPR construct. Red plant and or tissue indicates GE plant. Created with BioRender.com.

Constitutive promoters can be defined as those that lead to the continuous expression of its associated gene transcript in the cell, irrespective of environmental or developmental circumstances (Benfey and Chua, 1990). Promoters used in plant biotechnological approaches can be divided into three different sub‐classes: viral, bacterial and plant constitutive promoters, based on their origin (Kummari *et al*., 2020). Among virus‐derived promoters, the *Cauliflower Mosaic* virus 35S (*CaMV35S*) promoter (p35S) is widely employed in the creation of genetically modified organisms (GMOs) and for augmenting gene expression in various plant species (Amack and Antunes, 2020; Hull *et al*., 2009; Shcherbak *et al*., 2023; Shi *et al*., 2021; Wu *et al*., 2022). Indeed, the *CaMV35S* promoter, or its variants, is present in over 60% of all transgenic crops cultivated globally (Amack and Antunes, 2020; Carpenter *et al*., 2002). While this promoter exhibits a high level of expression in eudicot flowering plants, its expression in monocots such as grasses is reduced (Pih *et al*., 1996). It stands out as the most extensively researched and prevailingly utilized promoter for constitutive gene editing in plants (CGE) for driving Cas9 expression in many crops (Figure 2a) (Jiang *et al*., 2013; Lee *et al*., 2023b; Saini *et al*., 2023; Villette *et al*., 2024). Apart from *CaMV35s* promoter, the *Cestrum Yellow Leaf Curling virus (CmYLCV)* promoter exhibits comparable or higher levels of expression than the p35S or *pZmUbi* in both monocots and dicots (Stavolone *et al*., 2003). The *CmYLCV* promoter has been effectively employed for the expression of polycistronic gRNAs in tomatoes, which indicates the versatility and efficacy of the *CmYLCV* promoter in facilitating the simultaneous expression of multiple gRNAs, thereby enabling efficient genome editing (Čermák *et al*., 2017). Additionally, a promoter derived from *Cassava Vein Mosaic* virus *(CsVMV)* has also been employed to drive the expression of Cas for GE in barley, *Brassica oleracea*, and Arabidopsis, and is reportedly more efficient for CRISPR activity compared to the *UBI10* promoter in Arabidopsis (Castel *et al*., 2019; Lawrenson *et al*., 2015). In addition to viral‐based promoters, constitutive bacterial promoters like *Nopaline synthase* (*pNos*), *Octopine synthase* (*pOcs*) and *Mannopine synthase* (*pMas*) promoters have been utilized for plant genetic engineering (Kummari *et al*., 2020). In plant GE system, these promoters are primarily utilized for the expression of a selection marker and reporter genes (Grutzner *et al*., 2021; Kobercová *et al*., 2023; Lu and Tian, 2022; Wang *et al*., 2023a), However, *pMas* has recently been used to drive Cas9 in *Populus* with an improved mutation rate up to 75% (An *et al*., 2021).

Besides viral and bacterial‐based promoters, plant‐origin constitutive promoters are commonly utilized to introduce desired traits in crop plants. For instance, rice *Actin 1* (*OsAct1*) (McElroy *et al*., 1991), maize *Alcohol Dehydrogenase1* (*ZmAdh1*) (Fromm *et al*., 1990) and maize *Ubiquitin 1* and *2* (*ZmUbi1* and *ZmUbi2*) (Christensen *et al*., 1992). Different plant *Ubiquitin* gene (*UBI/UBQ*) promoters have been widely used for GE as an alternative to the 35S promoter, and with higher efficiency (Lee *et al*., 2023a; Zhang *et al*., 2016b, 2019). In 2020, a comparative study was conducted to evaluate the efficacy of four different promoters, namely *Ubiquitin* promoter *pAtUBQ10*, egg‐cell specific *pAtEC1.2*, meiosis‐specific promoter *pAMGE3* and p35S for GE in Arabidopsis. The study revealed that Cas9 driven by the *Arabidopsis thaliana UBQ10* promoter significantly enhanced the efficiency of GE by 95% in Arabidopsis and by 70% in *Medicago truncatula* (Wolabu *et al*., 2020). Moreover, Kurokawa *et al*. (2021) reported that the parsley *Ubiquitin* promoter (*PcUBI*) significantly enhanced gene‐editing efficiency by driving the expression of SpCas9 compared to the *pCaMV35S*, ribosomal protein S5 A promoter (pRPS5A) and *pZmUBI* in the Arabidopsis genome, particularly under heat stress conditions. Furthermore, two switchgrass ubiquitin promoters, *Panicum virgatum Ubiquitin 1* (*PvUbi1*) and *ubiquitin 2* (*PvUbi2*) have been identified and evaluated for transient and stable expression across a range of plant species and tissues (Mann *et al*., 2011). The efficacy of the *PvUbi1* promoter has been evaluated in wheat and barley for driving the expression of sgRNA and has been demonstrated to be equally efficient as *CmYLCV* promoter, hereby highlighting its potential utility as a reliable tool for precise genome editing in this important cereal crop species (Čermák *et al*., 2017). Furthermore, the ubiquitin promoter from rice (*OsUbi*) has been used to drive the expression of Cas12b from *Alicyclobacillus acidoterrestris* (*AaC2c1*), enabling heat‐inducible GE in cotton (Wang *et al*., 2020a). A newly‐developed endonuclease, ISDra2, has also been expressed with the rice *OsUbi10* promoter, achieving a mean editing efficiency of 33.6% (Karmakar *et al*., 2024). The development of the engineered SpRY Cas9 variant has also enabled GE with strong constitutive promoters at relaxed PAM sites in rice and Dahurian larch, up to 79% and 72.5%, respectively, thereby breaking the PAM restriction barrier in plant genomes (Ren *et al*., 2021b). Additionally, two strong constitutive promoters from *Glycine max*—*GmUBI3* and *GmM4*—have been employed in a PAM‐less SpRY GE system, resulting in editing efficiencies of up to 57.7% (Chen *et al*., 2024).

Among plant *Ubiquitin* promoters, the *pZmUBI* has emerged as a popular choice in biotechnological applications for its robustness and efficacy in driving CRISPR/Cas‐mediated Cas expression across various monocot species (Lv *et al*., 2024; Massel *et al*., 2022; Wang *et al*., 2023b). In a more recent development, a plant ultra‐multiplex genome editing system enabled the targeting of multiple genes and genomic sites in plants. This system utilizes the Cas9 or dCas9 gene driven by *pZmUBI* promoters coupled with various sgRNA promoters to construct a rice knockout plasmid, with 49 sgRNA expression cassettes, resulting in high co‐editing efficiency (Wu *et al*., 2024). Promoter strength has been tested with a single transcriptional unit (STU) CRISPR‐Cas9 and STU‐Cas 2.0 system where *pZmUBI* drove 8% and 20% higher mutagenesis for two target sites within the rice *Phytoene Desaturase* gene *(OsPDS)* compared to the p35S promoter. This demonstrated robust GE efficiencies, suggesting that *pZmUBI* may be a more reliable choice for GE in monocot species, or at least in some cereals (Tang *et al*., 2016, 2019). In addition to STU, a multiple single transcript unit surrogate reporter (STU‐SR) and single‐strand annealing (SSA) system has been successfully developed to improve the selection of gene‐edited plants. This system has been tested with both Cas proteins and variants like PAM‐less SpRY and achieved a 33.3% increase in editing efficiency in the case of *pZmUBI*‐driven STU‐SR‐SSA system (Ren *et al*., 2021b; Tang *et al*., 2024). Additionally, this promoter proved to be highly versatile for driving the expression of various other Cas proteins, including, xCas9‐3.7 (Zhong *et al*., 2019), Cas12j2 (Liu *et al*., 2022a), Cas12a (Li *et al*., 2018; Tang *et al*., 2017; Zheng *et al*., 2023). Furthermore, nine Cas12a orthologs were tested for GE under the control of *pZmUBI*, where six of them (ErCas12a, Lb5Cas12a, BsCas12a, Mb2Cas12a, TsCas12a and MbCas12a) exhibited increased editing activity in rice (Zhang *et al*., 2021). Additionally, IsDge10 has been tested in rice protoplasts with *pZmUBI* in combination with *OsUbi1* promoter, as a dual Pol II system for multiplexed GE with editing efficiencies ranging from 4.3% to 18.2% (Zhang *et al*., 2024b). These findings highlight the versatility of *pZmUBI* in driving different Cas variants and enhancement of GE efficiency in plants.

Apart from the employment of constitutive heterogenous promoters, utilization of species‐specific plant ubiquitin promoter *VvUBQ2* for GE with CRISPR/Cas, significantly increases the transcript abundance of Cas9 in grapevine, when compared to p35S (Ren *et al*., 2021a). Description of constitutively expressed promoters utilized for plant GE are listed in Table 1.

**Table 1:** Types of Pol II promoters utilized for plant GE

Types of promoters	Name of promoters	Source species	Host species	Expression	Mutation rate (%)	References
Constitutive promoters	35S	*Cauliflower Mosaic Virus (CaMV)*	*Arabidopsis thaliana*	Cas9	22.1–24.3	Zhang *et al*. (2017a)
			*Citrus sinensis*		11.5–64.7	Peng *et al*. (2017)
			*Citrus paradisi*		23–89	Jia *et al*. (2017)
			*Petunia hybrid*		55.6–87.5	Zhang *et al*. (2016a)
			*Populus*		100	Zhou *et al*. (2015)
			*Populus tomentosa*		51	Fan *et al*. (2015)
			*Nicotiana benthamiana*		1.8–2.4	Nekrasov *et al*. (2013)
			*Solanum lycopersicum*		48	Brooks *et al*. (2014)
			*Arabidopsis thaliana*	STU‐Cas9	9.1 to 19.9	Tang *et al*. (2016)
			*Nicotiana tabacum*		38.1 to 65	
			*Oryza sativa*		9	
	2X35S	*Engineered*	*Arabidopsis thaliana, Nicotiana benthamiana*	Cas9	5–10	Ordon *et al*. (2017)
			*Citrullus lanatus*		100	Tian *et al*. (2017)
			*Gossypium hirsutum*		98.8–100	Li *et al*. (2017b)
			*Salvia miltiorrhiza*		11.5–30.8	Li *et al*. (2017a)
			*Solanum tuberosum*		NA	Wang *et al*. (2015b)
			*Nicotiana tabacum*		81.8–87.5	Gao *et al*. (2015a)
	BiP 35S		*Oryza sativa*	Cas9	20.7–52.9	Ren *et al*. (2019)
	Larix Promotor	*Larix gmelinii*	*Larix gmelinii*	SpRY	72.5	Ren *et al*. (2021b)
			*Oryza Sativa*		62.5	
	*PvUbi1, PvUbi2*	*Panicum virgatum*	*Panicum virgatum, Oryza sativa and Nicotiana tabacum*	gRNA	NA	Mann *et al*. (2011)
	*PcUbi*	*Petroselinum crispum*	*Solanum lycopersicum, Oryza sativa*	Cas9	4.3–18.3	Shimatani *et al*. (2017)
			*Arabidopsis thaliana*		26.70	Fauser *et al*. (2014)
	*ZmUbi*	*Zea mays*	*Zea mays*	Cas9	100	Xing *et al*. (2014)
			*Zea mays*		77–100	Svitashev *et al*. (2015)
			*Triticum aestivum*		12.9–20.7	Gil‐Humanes *et al*. (2017)
			*Triticum aestivum*		5.60	Wang *et al*. (2014)
			*Hordeum vulgare*		10–23	Lawrenson *et al*. (2015)
			*Oryza sativa*		85.40	Ma *et al*. (2015)
			*Arabidopsis thaliana*	STU‐Cas9	2.7–8.9	Tang *et al*. (2016)
			*Nicotiana tabacum*	STU‐Cas9	19.4–59.4	
			*Oryza sativa*	STU‐Cas9	30	
			*Oryza sativa*	STU‐Cas9 2.0 (STU‐Cas9‐RZ)	74.5	Tang *et al*. (2019)
				STU‐Cas9 2.0 (STU‐Cas9‐Csy4)	88.2	
				STU‐Cas9 2.0 (STU‐Cas9‐tRNA)	81	
				STU‐Cas12a	20	
			*Oryza sativa*	STU‐SR‐SSA‐SpRY	33.3–53.8	Tang *et al*. (2024)
			*Oryza sativa*	STU_SR‐SSA‐Cas9	28.5–100	
			*Oryza sativa*	IsDge10	4.3 to 18.2	Zhang *et al*. (2024b)
			*Oryza sativa*	LbCas12a	10–35	He *et al*. (2024)
			*Oryza sativa*	Cas12a (ErCas12a, Lb5Cas12a, BsCas12a, Mb2Cas12a, TsCas12a and MbCas12a)	Nearly 100	Zhang *et al*. (2021)
			*Oryza sativa*	Cas9	5–80	Zhong *et al*. (2019)
			*Oryza sativa*	LbCas12a	64.4	He *et al*. (2024)
			*Oryza sativa*	Mb2Cas12a	60–75	
	*OsUbi*	*Oryza sativa*	*Oryza sativa*	Cas9	Up to 100	Xie *et al*. (2015)
		*Oryza sativa*	*Gossypium hirsutum*	Cas12b (AaC2c1)	6.34–98.68	Wang *et al*. (2020a)
	*GmUBI3*	*Glycine max*	*Glycine max*	SpRY	Up to 57.7	Chen *et al*. (2024)
	*GmM4*	*Glycine max*	*Glycine max*	SpRY	Up to 57.7	
	*OsUbi10*	*Oryza sativa*	*Oryza sativa*	TnpB(ISDra2)	33.58	Karmakar *et al*. (2024)
	*AtUBQ*	*Arabidopsis thaliana*	*Arabidopsis thaliana*	Cas9	13–93	Zhang *et al*. (2016b)
	*AtUBQ10*	*Arabidopsis thaliana*	*Arabidopsis thaliana*	Cas9	95	Wolabu *et al*. (2020)
			*Medicago*		70	
	*VvUBQ2*	*Vitis vinifera*	*Vitis vinifera*	Cas9	10.51–20.85	Ren *et al*. (2021a)
	*MdUBQ10*	*Malus domestica*	*Malus domestica (Protoplast)*	Cas9	NA	Wang *et al*. (2021)
	*SiUBI*	*Setaria italica*	*Zea mays*	Cas9	3.2–7.4	Barone *et al*. (2020)
Tissue specific promoters						
Meristematic and germline specific	*EF1*	*Arabidopsis thaliana*	*Arabidopsis thaliana*	Cas9	32.80	Osakabe *et al*. (2016)
Egg cell‐specific	*EC1.2*	*Arabidopsis thaliana*	*Arabidopsis thaliana*	Cas9	2.5–6.2	Ryder *et al*. (2017)
	*EC1.2e1.1p*	*Arabidopsis thaliana*	*Arabidopsis thaliana*	Cas9	8.3–42.9	Zheng *et al*. (2020)
			*Glycine max*	Cas9	26.80	
	*DD45*	*Arabidopsis thaliana*	*Arabidopsis thaliana*	Cas9	25–30 fold higher than UBI promoter	Ordon *et al*. (2020)
Developing flower specific	*AP1*	*Arabidopsis thaliana*	*Arabidopsis thaliana*	Cas9	NA	Gao *et al*. (2015b)
Dividing cell‐active specific	*ICU2*	*Arabidopsis thaliana*	*Arabidopsis thaliana*	Cas9	90.00	Hyun *et al*. (2015)
	*YAO*	*Arabidopsis thaliana*	*Arabidopsis thaliana*	Cas9	90.50	Yan *et al*. (2015)
	*RPS5A*	*Arabidopsis thaliana*	*Arabidopsis thaliana*	Cas9	100	Tsutsui and Higashiyama (2017)
Pollen specific	*PLIM2bP*	*Gossypium hirsutum*	*Gossypium hirsutum*	Cas9	3.29–6.45	Lei *et al*. (2021)
	*MYB24P*	*Gossypium hirsutum*	*Gossypium hirsutum*			
Male and female germline specific	*SPL*	*Arabidopsis thaliana*	*Arabidopsis thaliana*	Cas9	37% higher than UBI promoter	Mao *et al*. (2016)
Root cap specific	*SMB*	*Arabidopsis thaliana*	*Arabidopsis thaliana*	Cas9	51–92	Bollier *et al*. (2021)
Meiosis‐specific	*DMC1*	*Arabidopsis thaliana*	*Arabidopsis thaliana*	Cas9	37–64	Xu *et al*. (2018)
Root specific	*GCS*	*Arabidopsis thaliana*	*Glycine max, lotus japonicus, Solanum lycopersicum*	Cas9	76.70	Liu *et al*. (2022b)
Synthetic tissue specific	pTissue‐Specific/pOp6‐min35s	NA	*Arabidopsis thaliana*	Cas9	NA	Gehrke *et al*. (2023)
	pTissue‐specific/LexA‐min35s	NA	*Arabidopsis thaliana*	Cas9	59–73	Wang *et al*. (2020b)
Inducible promotors						
Virus	rgsCaM	*Solanum lycopersicum Yellow Leaf Curl Virus*	*Solanum lycopersicum*	Cas9	NA	Ghorbani Faal *et al*. (2020)
	*pV86*	*Beet severe curly top virus (BSCTV)*	*Nicotiana benthamiana*	Cas9	41	Ji *et al*. (2018)
	*pC86*				99	
Oestrogen	ip35S	*Cauliflower Mosaic Virus*	*Arabidopsis thaliana*	Cas9	59–73	Wang *et al*. (2020b)
	*IpWOX5*	*Arabidopsis thaliana*	*Arabidopsis thaliana*	Cas9	52–70	
	*XVE Promotor*		*Oryza sativa*	STU‐Cas9	14	Tang *et al*. (2016)
	*XVE Promotor*		*Oryza sativa*	Cas9	20	
Heat	*GmHSP17.5*	*Glycine max*	*Oryza sativa*	Cas9	50–63	Nandy *et al*. (2019)
	*HSP18.2 p*	*Arabidopsis thaliana*	*Arabidopsis thaliana*	Cas9	95–100	Hu and Yu (2022)
	*Hsp26*	*Zea mays*	*Zea mays*	Cas9	3.2–99	Barone *et al*. (2020)
Synthetic heat inducible	*HSE‐COR15A*	NA	*Arabidopsis thaliana*	Cas9	11.13–12.42	Liang *et al*. (2023)

Although constitutive promoters are commonly preferred for plant CGE, their indiscriminate activation may disrupt the organism's cellular machinery, interfere with reproduction and development and potentially induce toxicity through the constitutive ectopic expression of transgenes. They can also result in epigenetic silencing (Matsunaga *et al*., 2019; Vaucheret and Fagard, 2001). Furthermore, when expression of Cas9 is driven by constitutive promoters, it often leads to the formation of chimeras, necessitating screening for heritable homozygous mutants in subsequent generations (Feng *et al*., 2014; Singha *et al*., 2021). Moreover, inheritance of the mutation is limited as even so‐called constitutive promoters are often much less active in gametes, for example, in soybeans (Zheng *et al*., 2020). Additionally, constitutive promoters make it challenging to study genes that are specific to organs or tissues without disturbance on neighbouring cells. Overcoming these challenges mandates the precise utilization of native tissue/organ specific, induced (biotic and abiotic stress/hormones/chemicals/plant growth regulators/light/others) and synthetic promoters for effective GE.

## Tissue‐specific and inducible promoters for spatiotemporal GE in plants

The expression level of specific genes is finely tuned by tissue‐specific (TSPs), and inducible promoters in a spatiotemporal manner. The specific pattern of expression for TSPs and inducible promoters varies depending on the promoter sequence and inputs such as tissue specificity, developmental stage, environmental conditions and hormonal cues, among others. These promoters can be harnessed to fine‐tune desirable agronomic traits and to cultivate crops with resilience to diverse stresses, improved nutritional values and increased productivity with reduced pleiotropic effects of constitutive promoters (Jeong and Jung, 2015). TSPs allow the study of genetic elements within precise cellular, tissue or organ while minimizing interference with other cell or tissue types, or organs. Many TSPs have been characterized and only a few have been evaluated for potential GE applications to date, indicating their future potential utilization for plant GE (Alotaibi *et al*., 2018; Backiyarani *et al*., 2022; Chen *et al*., 2023; Dasgupta *et al*., 2020; Li *et al*., 2019, 2023b; Roh *et al*., 2021).

The use of TSPs for GE in plants has been promoted as it avoids some of the main challenges associated with CGE (Figure 2b). For instance, the highly root‐cap‐specific promoter, *SOMBRERO/ANAC033* (*pSMB*), has been successfully utilized to drive the expression of Cas9 specifically within the root cap of Arabidopsis, enabling tissue‐specific knockout (TSKO) (Table 1) (Decaestecker *et al*., 2019). More recently, Arabidopsis root‐specific promoter, *Gamma Glutamyl cysteine Synthetase* (*pAtGCS*) has also been identified and pAtGCSpro2411‐Cas9 system demonstrated an impressive efficiency in producing homozygous/biallelic mutants, with a success rate of 76.7% compared to 56.7% observed for the p× 35Spro‐Cas9 system (Liu *et al*., 2022b). Additionally, the tomato shoot apical meristem and root tip‐specific *Elongation factor‐1 α* gene (*pSlEF1α*) promoter is effective in driving Cas9 expression for stable genome GE in tomato and soybean (Hashimoto *et al*., 2018; Niu *et al*., 2023), and in *Salvia miltiorrhiza* for transient GE screening (Shao *et al*., 2024). Moreover, heritable GE specific to fruit has been achieved in tomato using the fruit‐specific promoter *Phosphoenolpyruvate Carboxylase 2 (pPPC2)* to drive CRISPR‐Cas9 expression; this approach successfully mitigated pleiotropic effects observed when using constitutive promoter (Feder *et al*., 2020). In addition to root and fruit‐specific promoters, two pollen‐specific promoters in cotton, from the genes encoding an R2R3‐MYB transcription factor (*pGhMYB24*) and a LIM‐domain protein (*pGhLIM2b*) have been successfully utilized for Cas9 expression to edit endogenous genes in pollen (Table 1) (Lei *et al*., 2021).

To mitigate the challenges of heritable mutant generation by constitutive promoters, germline‐specific promoters have been employed for TSGE, and have been found to significantly enhance the frequency and heritability of mutations in plants while mitigating the formation of somatic chimeras (Table 1) (Rahman *et al*., 2022; Zhou *et al*., 2023b). For example, Arabidopsis egg cell‐specific *EC1.2* gene promoter was used to drive CRISPR/Cas9 to generate homozygous mutants in a single generation with editing in multiple target genes in Arabidopsis (Wang *et al*., 2015c). Furthermore, the Ribosomal protein S5 A (RPS5a) and egg cell‐specific *downregulated in dif1 45 (pDD45)* promoters have been utilized for genome editing in Arabidopsis, resulting in significantly increased editing efficiency. These promoters have demonstrated a 20‐ to 30‐fold enhancement in editing efficiency, with high frequencies achieved within a single generation, compared to the ubiquitin promoter (Ordon *et al*., 2020). In more recent studies, *DD45* promoter‐driven Cas9 has also been employed for precise gene targeting in Arabidopsis with multiple gRNAs (Li *et al*., 2024). Another egg‐cell specific promoter, *pAtEC1.2e1.1* has been used for Cas9 expression in Arabidopsis and soybean with mutation frequencies of 8.3%–42.9% and 26.8%, respectively, while it is noteworthy that high somatic mutations were observed in hairy roots with a constitutive promoter in soybean (Zheng *et al*., 2020). Nevertheless, Cas9 driven by the Arabidopsis *YAOZHE (pYAO)* promoter has been utilized to significantly enhance GE in Arabidopsis (Yan *et al*., 2015), and in many crops such as citrus (Zhang *et al*., 2017b), sweet orange (Tang *et al*., 2021) and cassava (Wang *et al*., 2022c). This promoter has been proven to increase mutagenesis fivefold in somatic tissues and up to 100‐fold in the germline cells under heat treatment in Arabidopsis by increased activity of *SpCas9* (LeBlanc *et al*., 2018). This also highlights the potential of promoters to enhance targeted mutagenesis in plants using CRISPR/Cas9 when combined with abiotic stress or other environmental cues. In addition to TSGE by germline‐specific promoters, many other promoters have also been utilized (Table 1) for CRISPR/Cas expression in Arabidopsis, such as vessel‐specific secondary wall NAC binding element promoter (*pSNBE*) for the reduction of lignin (Yu *et al*., 2021), cell‐type‐specific promoters such as (*NST3*/*SND1*) for secondary cell wall development in fibre cells (Liang *et al*., 2019), *Wuschel‐*related homeobox promoter *(pWOX5)*, *CyclinD6‐1* promoter *(pCYCD6‐1)* and *Endodermis7* promoter *(pEN7*) for cell‐type‐specific genetic manipulation in Arabidopsis roots (Li *et al*., 2023a).

In addition to TSPs, the availability of various inducible promoters has paved the way for induced gene editing (IGE) in response to various biotic and abiotic stimuli (Figure 2c), such as virus‐induced GE with the regulator of gene silencing calmodulin promoter from tomato (pRGSCaM) (Ghorbani Faal *et al*., 2020), two *Beet Severe Curly Top* virus *(pBSCTV)* induced promoters, *pV86* and *pC86* in Tobacco (Ji *et al*., 2018), heat‐inducible promoter, Heat‐shock protein 18.2 (pHSP18.2) from *Arabidopsis thaliana* (Liang *et al*., 2023) and a *Heat shock protein26* promoter (p*Hsp26*) from maize (Table 1) (Barone *et al*., 2020). In maize, Hsp26 promoter‐driven Cas9 activity has been compared with the constitutive ubiquitin promoter from *Setaria italica (pSiUbi)* which showed gene targeting efficiency of 18% and 3.2%, respectively, which indicates that gene targeting with higher efficiency can be achieved through IGE (Table 1) (Barone *et al*., 2020). Similarly, the heat shock inducible CRISPR/Cas9 (HS‐CRISPR/Cas9) system has been evaluated in rice using *heat‐shock protein 17.5E (pHSP17.5E)* gene promoter from soybean to express Cas9. In comparison to the strong constitutive rice *ubiquitin* promoter (*pRUBI*), the HS‐CRISPR/Cas9 system achieved mutations at a rate of ≥50%, with negligible off‐target effects (Nandy *et al*., 2019), which indicates the IGE system is a promising tool for improving precision with limited or no off‐targets for plant GE. Additionally, Inducible BiPs have also been identified that could be utilized for plant GE, for instance, light‐inducible BiP (Zhu *et al*., 2023a). Indeed, the utilization of TSPs and inducible promoters provides significant opportunities for achieving spatiotemporal GE in plants.

## Unlocking high precision in plant GE through synthetic promoters

The diverse array of naturally occurring promoters within plant genomes has been a subject of profound interest (Brooks *et al*., 2023; Porto *et al*., 2014; Vedel and Scotti, 2011) and many bioinformatic tools for identifying promoter and regulatory elements have been developed. Tools such as PlantTFDB 3.0 (Jin *et al*., 2014), TSSPlant (Shahmuradov *et al*., 2017), PlantPAN3.0 (Chow *et al*., 2019), Cr‐Prom, specifically for rice (Shujaat *et al*., 2021) and iProm‐Zea (Kim *et al*., 2022), are available for analysing promoters and DNA motifs. Yet, our understanding of all regulatory elements and their interactions remains incomplete, leaving many promoters insufficiently characterized (Cazier and Blazeck, 2021; Fan *et al*., 2023). Several factors contribute to the challenge of fully understanding promoter functions in plants. First, defining promoters accurately is often elusive, requiring precise delineation of their boundaries and core elements, which vary not only among different plant species but also among genes within the same species (Andersson and Sandelin, 2020). Furthermore, the interactions and dynamics between promoter elements (such as enhancers and silencers) and TFs, as well as other regulatory elements like non‐coding RNAs, are complex and not fully understood (Wang *et al*., 2022a). Epigenetic modifications also play a significant role, as they can have profound but diverse effects on gene expression, complicating the characterization of tissue and environmentally specific promoter activities (Gibney and Nolan, 2010). Additionally, methodological limitations in capturing these interactions under various physiological conditions hinder progress. However, emerging technologies like single‐cell sequencing (Preissl *et al*., 2023) and CRISPR‐based transcriptional profiling (Wang *et al*., 2022b), along with advanced integrative data analysis and the application of artificial intelligence algorithms (Lai *et al*., 2019; Sganzerla Martinez *et al*., 2023) hold promise for providing deeper insights.

**Figure 3:**
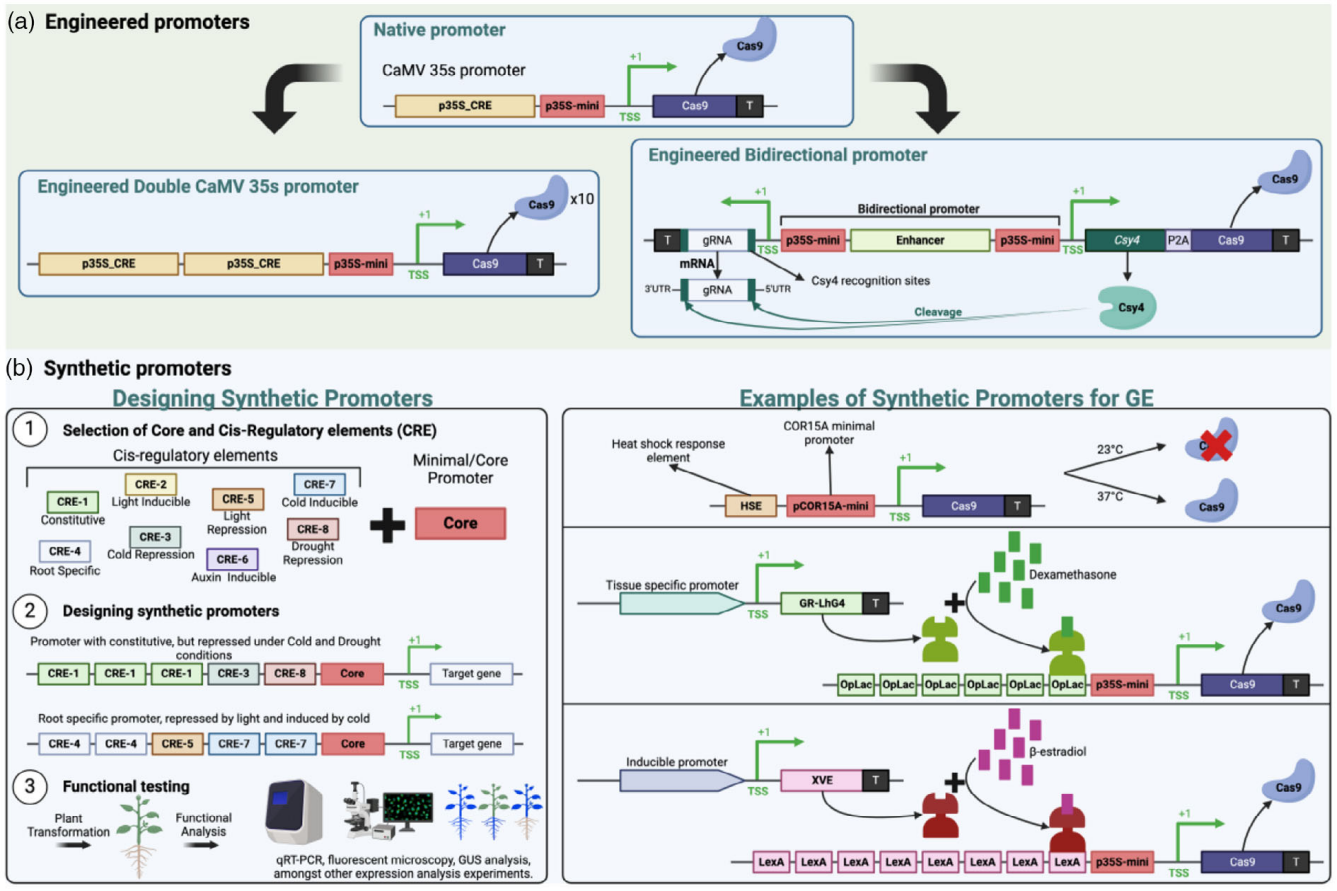
Examples of engineered and synthetic promoter for GE in plants. (a) Example of CaMV 35S engineered promoters utilized for plant GE. (b) Designing and Examples of synthetic promoter utilized for GE in plants. CRE: Cis‐regulatory element; p35S: CaMV 35S promoter; p35S‐mini: Minimal/Core p35S; Cas9: CRISPR‐associated protein 9; TSS: Transcription Start Site; T: Transcription terminator; gRNA: guide RNA; *Csy4*: gene encoding bacterial endoribonuclease Csy4; P2A: 2A peptide sequence for ribosomal skipping; GR‐LhG4: ligand‐binding domain of a glucocorticoid receptor fused to the amino terminus of the synthetic transcription factor LhG4;OpLac: optimized *lac* operator sequence: XVE: Synthetic transcription factor assembled by fusion of the DNA‐binding domain of the bacterial repressor LexA (X), the acidic transactivating domain of VP16 (V) and the regulatory region of the human oestrogen receptor (E; ER). Created with BioRender.com.

Engineered and synthetic promoters have emerged as strategic tools to enhance this diversity in a controlled manner while fine tuning the spatiotemporal expression of the genes under control (Jores *et al*., 2021; Liu and Stewart, 2016; Yasmeen *et al*., 2023). In this context, we will refer to ‘Engineered promoters’ as naturally occurring promoters that have been subjected to targeted modifications (Mehrotra *et al*., 2011; Pandiarajan and Grover, 2018), rather than entirely artificial promoter sequences. These modifications may involve the selective elimination, alteration or addition of cis elements without fundamentally changing the primary structure or identity of the promoter (Figure 3a). Conversely, ‘synthetic promoters’ denote fully synthetic entities created *de novo* (Brophy *et al*., 2022; Jores *et al*., 2021). These promoters typically integrate the minimal promoter from a naturally occurring source with an arrangement of cis‐elements derived from various non‐related promoters (Figure 3b). Various strategies, including site‐directed/targeted mutagenesis (Huttanus *et al*., 2023), promoter editing (Shi *et al*., 2023), traditional cut‐and‐paste cloning with type II restriction enzymes (Clark *et al*., 2019), Gateway cloning (Hartley *et al*., 2000), Gibson assembly (Gibson *et al*., 2009), Golden Gate‐based DNA assembly systems (Engler *et al*., 2014; Hahn *et al*., 2020) and DNA synthesis (Yasmeen *et al*., 2023), can be employed to generate engineered or synthetic promoters. The selection of methods or combination strategies for generating synthetic promoters ultimately depends on the preferences of the user, the source of regulatory elements and the intended purpose of the synthetic promoters. Taken together, these approaches are expected to enable more precise manipulation of gene expression, offering researchers and breeders a potent tool (Yasmeen *et al*., 2023).

The use of engineered and synthetic promoters in GE promises significant advantages such as:

1. The use of minimal or short synthetic promoters with simple regulation patterns, compared to native plant promoters, which generally possess complex patterns spread across hundreds to thousands of nucleotides (Aysha *et al*., 2018; Bhullar *et al*., 2003; Cai *et al*., 2020);
2. Improved tissue specificity and control over the inducibility of the promoters, which addresses challenges such as unintended expression leaks, while providing unprecedented control over expression within tissues and temporal windows (Belcher *et al*., 2020; Jameel *et al*., 2020; Kumar *et al*., 2012; Lv *et al*., 2009; Mohan *et al*., 2017; Wang *et al*., 2015a);
3. Likewise, engineered and synthetic promoters can alleviate gene silencing by amalgamating functionally equivalent cis‐elements from various non‐plant organisms, thereby maximizing sequence diversity and contributing to the mitigation of gene‐silencing effects (Belcher *et al*., 2020; Yasmeen *et al*., 2023);
4. Engineering of promoters can also allow the generation of *de novo* synthetic gene circuits for controlling multiple traits in different tissues or response to different environmental or human‐driven signals for differential GE of multiple genes (Brophy *et al*., 2022; Pierre‐Jerome *et al*., 2016).

One of the first and most widespread examples of an engineered promoter is the double *CaMV 35S* promoter (p2x‐35S), which is based on the naturally occurring *CaMV35S* promoter with a duplication of the transcription‐activating sequences upstream of the TATA box (Kay *et al*., 1987). This led to a 10‐fold increase in the expression compared to the native promoter (Kay *et al*., 1987) and has been used for multiple purposes over the last 35 years, such as the generation of transgenic plants and GE (Table 1). More recently, Tripathi *et al*. (2023) demonstrated the use of the Cas‐CLOVER system, driven by the p2x‐35S promoter, to target the *phytoene desaturase* gene in bananas. Another example of an engineered version of *CaMV35s* promoter for GE is the use of the bidirectional double‐mini 35S promoter, which consists of two *CaMV35S* minimal (p35S‐mini) promoters flanking an Arabidopsis enhancer region in opposite directions (Xie *et al*., 2001). Thus, this bidirectional double‐mini 35S promoter can direct the expression of both the Cas9 protein and the gRNA (Ren *et al*., 2019). As the use of bidirectional (BiP) promoters allows a single promoter to regulate the expression of two genes positioned on either side, it minimizes the risk of transcriptional gene silencing due to multiple copies of the same or similar promoters (Xie *et al*., 2001). Recently, the newly synthesized RNA Pol II promoter, named *Larix* promoter, developed from Dahurian larch, has been used to enable genome editing in gymnosperms via the PAM‐less SpRY system, achieving an average editing efficiency of 72.5%. This represents a promising approach for genome editing across diverse plant species, including gymnosperms (Ren *et al*., 2021b).

Synthetic promoters for inducible expression of the Cas9 protein have also been reported. For instance, the synthetic heat‐inducible promoter HSE‐COR15A, composed of the heat shock‐response element HSE and the minimal promoter from COR15A, has been recently tested for conditional GE under heat stress by temporally driving the expression of Cas9 (Liang *et al*., 2023). Likewise, the use of six 52 bp *lac* operators upstream p35S‐mini (pOp6) allowed the tunable expression of Cas9 in Arabidopsis (Gehrke *et al*., 2023). Thus, by using a dexamethasone inducible system GR‐LhG4/pOp6 (Craft *et al*., 2005) along with tissue‐specific expression of GR‐LhG4 synthetic transcription factor, it was possible to get precise spatiotemporal control of cas9 and therefore, GE (Gehrke *et al*., 2023). A similar example is the use of a synthetic oestrogen‐inducible promoter which consists of copies of XVE‐binding sites (LexA operator sequence) fused with p35s‐mini (pLexA) (Wang *et al*., 2020b). The chimeric transcriptional activator XVE (Siligato *et al*., 2016; Zuo *et al*., 2000) driven by constitutive, tissue‐specific or inducible promoters, provides an efficient strategy to conditionally knocked out genes in plants (Wang *et al*., 2020b). Additionally, conditional expression of STU CRISPR‐Cas9 has been tested with pXVE targeting the *OsPDS* gene in rice. The addition of oestrogen to transformed protoplasts resulted in significant targeted mutagenesis, though lower levels of mutagenesis were also observed in uninduced samples which were confirmed to be due to leaky expression of pXVE. Nevertheless, this demonstrates that the STU system can be conditionally regulated (Tang *et al*., 2016). More recently, efficient regulation of LbCas12a‐mediated GE has also been demonstrated using a pXVE::ACrVA1 system to control the expression of anti‐CRISPR, AcrVA1 with GE efficiency ranging from 10% to 35% (He *et al*., 2024).

The emergence of CRISPR activation (CRISPR‐a) tools has also substantially broadened the applicability of CRISPR methodologies for gene regulation (Chavez *et al*., 2015; Pan and Qi, 2023; Papikian *et al*., 2019; Xiong *et al*., 2021), and IGE systems can be rapidly accelerated by combining multiple synthetic biology strategies. One such example includes a light‐inducible synthetic optogenetic promoter, developed with plant‐usable light‐switch elements (PULSE), which is only activated in red light while remaining inactive in blue light. This promoter has been used for PULSE‐controlled expression of CRISPR–Cas9‐derived gene activators and plant TFs in Arabidopsis (Ochoa‐Fernandez *et al*., 2020). A copper‐inducible system has also been used for CRISPR/Cas‐based programmable transcription activation (PTA) in *Nicotiana benthamiana* by CBS:minDFR promoter, which contains four repeats of the copper binding site (CBS) operator assembled to the upstream of minimal promoter of the tomato NADPH‐dependent dihydroflavonol reductase (pDFR) (Garcia‐Perez *et al*., 2022). Additionally, synthetic, chemically inducible systems have been combined with CRISPR‐a to accelerate plant regeneration by activating morphogenic genes in both eudicots and monocots (Zhang *et al*., 2024a). Indeed, synthetic inducible systems hold significant potential to enhance plant GE more efficiently and precisely.

## Promoter editing for novel and improved trait development

Intriguingly, engineered and synthetic promoters can be used not only to foster GE in plants but also the other way around, as GE can be used to modify the function of native promoters. Synthetic‐directed evolution (SDE) has been used to discover and modify plant traits (Rao *et al*., 2021). This approach employs localized sequence diversification and selection pressure to successively evolve promoters, enhancing their suitability for specific functions and identifying the optimal sequence from a pool of randomly generated synthetic promoters which encompass the generation of multiple conceivable mutations for a given promoter. Thus, through multiple cycles of mutation, testing and selection, the final promoter will be quantitatively optimized (Liu *et al*., 2021; Oliva *et al*., 2019; Rao *et al*., 2021; Rodriguez‐Leal *et al*., 2019). New promoters could result in better expression of tissue‐specific target genes, in more robust expression under specific environmental conditions, or the ability or inability to respond to certain stimuli (Rao *et al*., 2021; Yasmeen *et al*., 2023).

Another effective use of promoter editing for crop improvement involves employing CRISPR technologies to modify regulatory regions in promoters that control gene expression, as a route to impacting agronomic traits. For example, Rodríguez‐Leal *et al*. (2017) used CRISPR/Cas9 to target the promoters of the *CLV3* and *WUSCHEL* (*WUS*) genes in tomatoes, which regulate key traits such as fruit size, inflorescence branching and plant architecture. Specifically, they targeted the CArG repressor element in the promoter of *WUS and* used multiple guide RNAs to target the 2‐kbp promoter region of *CLV3*, which affected various regulatory elements within this region. This editing led to varying numbers of locules in the fruit, directly influencing fruit size. This example highlights the potential of promoter editing to create alleles with subtle yet beneficial changes for precision breeding. Similarly, Oliva *et al*. (2019) demonstrated the effectiveness of CRISPR–Cas9 promoter editing for enhancing disease resistance in rice. By editing effector binding elements (EBEs) in the promoters of *SWEET11*, *SWEET13* and *SWEET14* genes, they generated rice‐edited lines with robust, broad‐spectrum resistance to bacterial blight caused by *Xanthomonas oryzae* pv. oryzae.

An innovative approach for introducing quantitative trait variation (QTV) in crops is the novel CRISPR–Cas12a promoter editing (CAPE) system (Zhou *et al*., 2023a). This CAPE system integrates a promoter‐focused approach with the CRISPR–Cas12a GE system. Unlike Cas9, Cas12a induces staggered‐end double‐strand breaks (DSBs), which lead to much larger deletions, making it a more suitable tool for destroying CREs and achieving effective promoter editing (Tang *et al*., 2017; Zetsche *et al*., 2015; Zhou *et al*., 2023a). CAPE was successfully demonstrated in rice by targeting the promoters of OsGBSS1 and OsGS3 to create QTV for starch content and grain size respectively. Additionally, CAPE was applied to the OsD18 promoter, which encodes gibberellin biosynthesis GA3 oxidase, to generate semi‐dwarfism without affecting grain yield (a quantitative Green Revolution trait) (Zhou *et al*., 2023a). Thus, the CAPE system represents an efficient and precise tool for generating desirable quantitative traits in crops.

In summary, promoter editing represents a novel approach in the field of plant GE, potentially offering a route to optimizing key traits. From specific CRISPR‐based promoter editing to CAPE and SDE, these strategies can provide control over gene expression without altering coding sequence. The advances highlighted here underscore the broad applicability of promoter editing to address challenges in crop production. Promoted engineering has the promise to enable the development of more resilient, high‐yielding and adaptive crops that are better equipped to meet future sustainability and food security challenges.

## RNA polymerase III promoters: trailblazers in elevating plant GE

**Figure 4:**
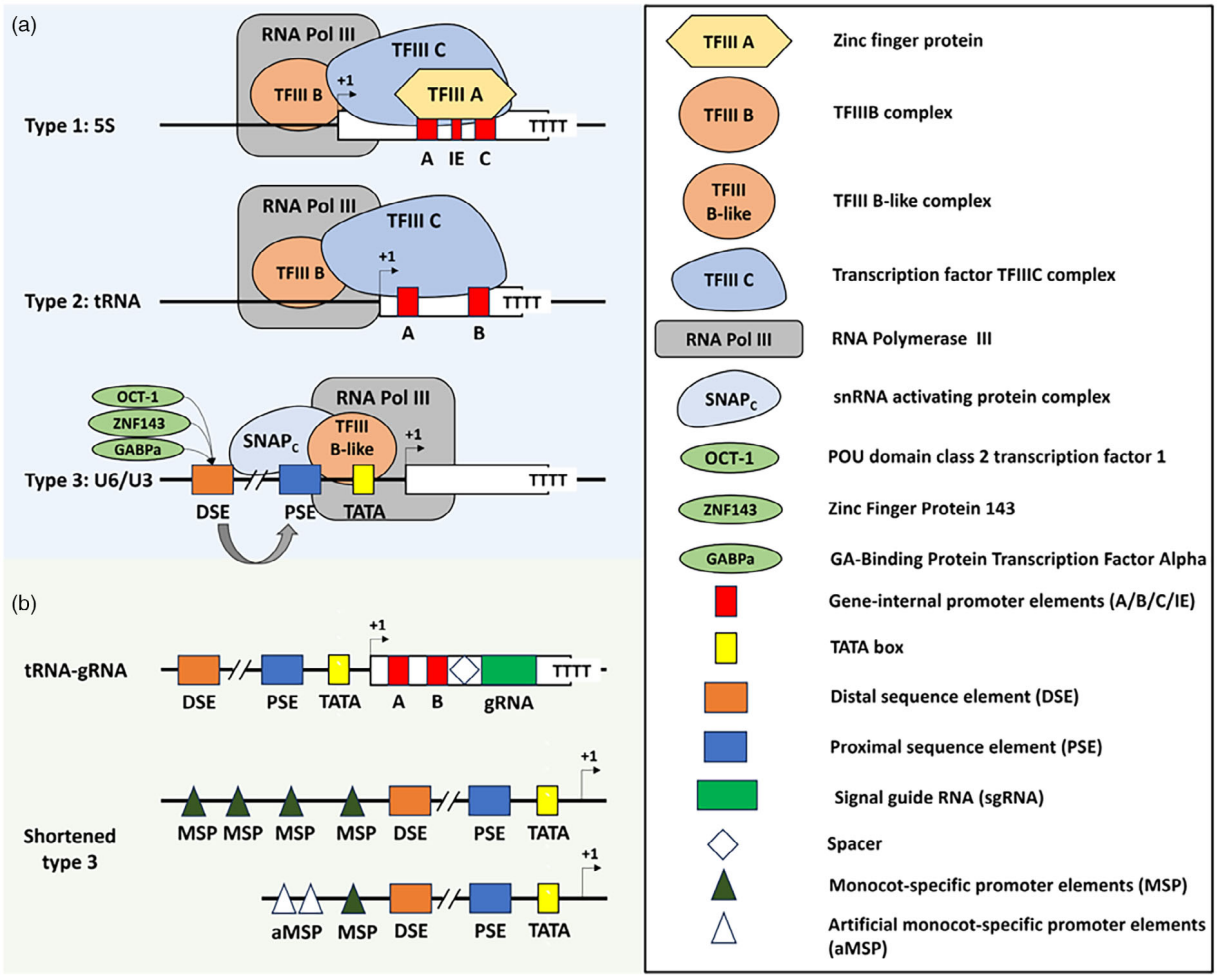
Schematic structure of RNA Pol III promoters. (a) Structure and pre‐initiation complex (PIC) of RNA Pol III promoters. Type 1 Pol III promoter: the gene‐internal promoter comprises A and C boxes separated by an intermediate element (IE). The zinc finger protein TFIIIA is first recruited followed by the TFIIIC complex and the TFIIIB complex then formed the PIC to recruit RNA Pol III. Type 2 Pol III promoter: the promoter is gene‐internal and consists of A and B boxes, which directly recruit TFIIIC. The PIC then assembles like on type 1 Pol III promoters. Type 3 Pol III promoter: the promoter contains a proximal sequence element (PSE), a distal sequence element (DSE) and a TATA box. DSE was recognized by regulatory factors such as POU domain class 2 transcription factor 1 (Oct‐1), zinc finger protein 143 (ZNF143/hStaf), and/or GA‐binding protein transcription factor alpha (GABPa). The PIC contains snRNA activating protein complex (SNAPc) bound to the PSE, and TFIII B‐like complex bound to the TATA box to recruit Pol III. (b) Structure of artificial RNA Pol III promoters. tRNA‐gRNA architecture consists of elements from type 2 and type 3 Pol III promoters (Xie *et al*., 2015). Shortened promoter: 5′ sequences deleted but retained one native MSP, the DSE, PSE and TATA box. Then added artificial MSPs to the 5′ region (Hao *et al*., 2020). Created with BioRender.com.

RNA Pol III is responsible for transcribing small non‐coding RNA molecules with a steady transcriptional activity acting on short, often tandemly repeated genes, including 5S ribosomal RNA (rRNA) genes, transfer RNA (tRNA) genes and some small nuclear RNA (snRNA) genes. Most RNAs transcribed by RNA Pol III remain in the nucleus instead of being transported to the cytoplasm (Hamm and Mattaj, 1990). Both initiation and termination of RNA Pol III transcription involve specific sequences (elements) and processes distinct from those of RNA Pol I and RNA Pol II. RNA Pol III promoters are divided into three categories, named types 1, 2 or 3 (Schramm and Hernandez, 2002). Type 1 (e.g. 5S rRNA gene promoters) and type 2 (e.g. tRNA gene promoters) contain elements necessary for transcription within the gene transcription region while type 3 promoters (e.g. snRNA *U6* and *U3* promoters) rely on elements upstream of the transcription start site (+1 TSS) (Figure 4a). Concordantly, the different Pol III promoter structures result in different preinitiation complex (PIC) components for RNA Pol III recruitment (Figure 4a). Interestingly, although diverse mechanisms partake in RNA Pol III transcription termination, poly‐T is the most prevalent termination signal (Arimbasseri *et al*., 2013; Nielsen *et al*., 2013; Xie *et al*., 2022).

The structure of Pol III type 3 promoters, which do not rely on any element inside the gene region, makes them prime candidates for ectopic expression of small RNAs. For this reason, endogenous snRNA promoters (*U3* and *U6*) are commonly used in the CRISPR genome‐editing system to drive the gRNA transcription (Massel *et al*., 2022; Ren *et al*., 2021a). The elements of *U3* and *U6* promoters include a conserved TATA box and the upstream sequence element (USE, including a proximal sequence element and a distal sequence element) which are necessary for RNA Pol III initiation (Schramm and Hernandez, 2002). Besides, the efficiency of *U3* and *U6* promoters in monocots is also determined by the monocot‐specific promoter element (MSP) located upstream to the USE, present in one to three copies (Figure 4b) (Hao *et al*., 2020; Marshallsay *et al*., 1992). In addition, due to the interspecies conservation of RNA Pol III and snRNA promoter, the use of snRNA promoters across species can be achieved, although their efficiency tends to be lower than that of the original promoter (Charrier *et al*., 2019; Ma *et al*., 2015; Zhang *et al*., 2022).

Multiple endogenous copies of *U3* and *U6* genes are common in plant species. These genes' promoter regions were divergent, leading to a different expression/editing efficiency. For instance, in grape (*Vitis vinifera*), four endogenous *U3/U6* promoters were identified (two *U3* promoters and two *U6* promoters), and the editing efficiencies with the *VvU3* promoters were slightly lower than those obtained with the *VvU6* promoters (Ren *et al*., 2021a). One possible reason is that the eukaryotic *U3* promoter prefers a 5′ adenine (A) for transcription initiation whereas the *U6* promoter recognizes a 5′ guanine (G), and the A or G at the 5′ end of gRNA could affect the editing efficiencies (Kim *et al*., 2017; Pan and Qi, 2023). Besides, *VvU6.2* performed better than *VvU6.1* (Ren *et al*., 2021a). Indicating that genome editing efficiency can be improved by screening for efficient endogenous *U3/U6* promoters (Di *et al*., 2019; Massel *et al*., 2022). The other RNA Pol III promoters that have been utilized for plant GE are listed in Table 2.

**Table 2:** Pol III promoters utilized for plant GE

Species	Pol III promoter	Cas expression	Efficiency (%)	References
*Gossypium hirsutum*	*AtU6‐29*	2X p35S::Cas9	Control	Long *et al*. (2018)
	*GhU6.3*		4–6 times higher mutagenesis than control	
*Glycine max*	*AtU6‐26*	p35S::Cas9	3.2–9.7	Sun *et al*. (2015)
	*GmU6‐10*		14.7–20.2	
*Glycine max*	*GmU6‐1*	Cas9	8.3–14.4	Di *et al*. (2019)
	*GmU6‐2*		6.5–7.3	
	*GmU6‐3*		5.2–20.5	
	*GmU6‐4*		7.3–21.3	
	*GmU6‐5*		2.1–3.2	
	*GmU6‐6*		8.4–16.7	
	*GmU6‐7*		5.6–18.9	
	*GmU6‐8*		12.1–26.6	
	*GmU6‐9*		9.6–17	
	*GmU6‐10*		14.9–28	
	*GmU6‐11*		14.7–20.5	
*Hevea brasiliensis*	*HbU6‐1*	p35S::Cas9	8.47–24.92	Dai *et al*. (2021)
	*HbU6‐2*			
	*HbU6‐3*			
	*HbU6‐4*			
	*HbU6‐5*			
*Lupinus albus*	*AtU6.1*	2X p35S::Cas9	0	Zhu *et al*. (2023b)
	*LaU6.2*		20	
	*LaU6.5*		6.25	
	*LaU6.6*		20	
	*LaU6.9*		25	
	*AtU3d*		72.73	
	*LaU3.1*		56.25	
	*LaU3.2*		80	
	*LaU3.4*		75	
	*LaU3.7*		50	
*Musa acuminat*	*OsU6a*	pUbi::Cas9	0.29	Zhang *et al*. (2022)
	*MaU6c*		0.80	
	*MaU6c*	pUbi::opCas9	1.20	
*Vitis vinifera*	*AtU6*	pUBQ2::spCas9	13.67	Ren *et al*. (2021a)
	*VvU3.1*		14.65	
	*VvU3.2*		16.74	
	*VvU6.1*		22.10	
	*VvU6.2*		21.68	
*Oryza sativa*	*OsU3*	2X p35S::Cas9 (different Cas9 variants tested)	0–53.3	Mikami *et al*. (2015)
	*OsU6*	2X p35S::Cas9 (different Cas9 variants tested)	45.6–80	
	*OsU3*	p35S:HPTIPubi:Cas9	84.90	Hao *et al*. (2020)
	*OsU6a*		82.50	
	*OsU6b*		81.50	
	*OsU6c*		78.90	

In addition, sequence modifications or enhancers can be introduced to improve Pol III type 3 promoters, and therefore, GE efficiency (Figure 4b). For instance, a modified tRNA sequence was introduced to form a tRNA‐gRNA architecture that further boosts gRNA production (Xie *et al*., 2015). This tRNA‐gRNA architecture is also applied to generating polycistronic gRNA, which are processed by the endogenous RNase P and RNase Z (Li *et al*., 2022; Schwartz *et al*., 2016; Xie *et al*., 2015). This indicates that the combined use of snRNA promoters and tRNA could be a strategy to create transcriptional enhancers for RNA Pol III type 3 promoters. In addition, plant *U3* and *U6* promoters generally require a conserved USE and TATA box for effective transcription and shortened *U3* and *U6* promoters (reduced from 380–740 to 170–223 bp) containing only the USE, TATA box and several added artificial MSPs have been developed for GE in rice (Hao *et al*., 2020). Moreover, *in vitro* evidence proves that the TATA box alone contains all the necessary information to initiate RNA Pol III transcription (Duttke, 2014; Mitchell *et al*., 1992). This suggests that artificial promoters could further boost GE efficiency, despite cloning the endogenous snRNA promoter for gRNA expression.

## Conclusions and future perspectives

Transcription is a fundamental cellular function involved in gene regulation. It is achieved through the sequence‐specific binding of TFs to their target promoters, where they can either off or on the transcription of a gene, as well as controlling the gene transcript levels. Likewise, CRE sequences play an indispensable role in regulating gene expression through various mechanisms to achieve either constitutive or spatiotemporal expression (Yaschenko *et al*., 2022). Understanding the intricacies of regulatory elements provides valuable insights into the application of different native promoters and synthetic promoters for plant GE. The appropriate selection of promoters for plant GE is a crucial step, requiring the strategic utilization of promoter elements. The choice of promoters is predominantly contingent upon the specific transgenes intended for expression (Smirnova and Kochetov, 2020). While the use of constitutive promoters has been proven as an effective approach for GE in a diverse array of genes and crops (An *et al*., 2021; Čermák *et al*., 2017; Lee *et al*., 2023b; Lv *et al*., 2024; Ren *et al*., 2021a), it nonetheless presents certain constraints, particularly when targeting genes crucial for key developmental or cellular stages at diploid or haploid levels, which can lead to the manifestation of lethal phenotypes (Feder *et al*., 2020; Matsunaga *et al*., 2019; Zheng *et al*., 2020; Zhou *et al*., 2023b). Moreover, the employment of constitutive promoters restricts the ability to conduct dynamic analyses in specific tissues or at a particular temporal point (Freeman *et al*., 2011; Singha *et al*., 2021).

In recent years, the advent of TSGE employing TSPs, for instance, in CRISPR‐TSKO, has presented novel opportunities for crop genetic engineering and fundamental research (Decaestecker *et al*., 2019). Successful applications of CRISPR‐TSKO include the tissue‐specific edition of essential genes such as *cyclin‐dependent kinases* (*CDKs*) in roots and *Phytoene Desaturases* 3 (*PDS3*) in stomata of Arabidopsis (Decaestecker *et al*., 2019). Likewise, the Cas9‐based fruit‐specific silencing of tomato set domain containing polycomb protein (SlEZ2) produced reductions in seed mass and number without pleiotropic phenotypes, such as reduction in lycopene level, altered fruit shape and ectopic organ development, as observed when constitutively silenced (Feder *et al*., 2020). These methodologies could prove particularly advantageous when employed for GE in germline cells, focusing on the modification of genes intricately associated with the cellular division processes of both mitosis and meiosis (Rahman *et al*., 2022; Singha *et al*., 2021; Zheng *et al*., 2020; Zhou *et al*., 2023b). Certainly, in addition to TSPs, the identification and isolation of inducible promoters have played a crucial role in advancing GE capabilities. By harnessing inducible promoters, the expression of CRISPR/Cas components can be activated or suppressed in a spatiotemporal manner in response to specific external signals or environmental cues, which allows the fine‐tuning of GE activities, minimizing off‐targets and maximizing efficiency (Barone *et al*., 2020; Nandy *et al*., 2019). Therefore, underscoring the prime role and the future applications of non‐constitutive promoters for precise tissue‐ and temporal‐specific systems.

Despite all recent advances regarding TSPs and CREs (Huang *et al*., 2021; Singha *et al*., 2021) the current characterization of these elements remains limited, which limits our ability to rationally design these regulatory elements for precision applications. One major limitation is the incomplete understanding of promoter dynamics and interactions, particularly how various CREs cooperate under different physiological contexts, hindering the predictability and consistency needed for targeted loci or motif‐based approaches (Schmitz *et al*., 2021). Additionally, the potential for leaky expression of promoters in other cell types poses a challenge, which may influence the precise editing of the targeted tissue (Franco‐Zorrilla *et al*., 2014; Khan *et al*., 2023; Yaschenko *et al*., 2022).

The emergence of synthetic promoters represents a promising approach to addressing the challenges associated with native promoters. Due to the modular nature of synthetic promoters, these synthetic promoters could be designed to respond to user‐defined inputs, providing a high degree of control over gene regulation in plants (Jores *et al*., 2021; Liu and Stewart, 2016; Shrestha *et al*., 2018; Yasmeen *et al*., 2023), thus offering a versatile platform for fine‐tuning gene expression based on specific requirements. Synthetic promoters can even drive expression at the single‐cell level, providing a level of precision, that might not easily be attainable with natural promoters (Yasmeen *et al*., 2023). The development and implementation of synthetic promoters contribute to advancing the sophistication and controllability of GE strategies, offering enhanced precision and adaptability in the intended gene edition (Kar *et al*., 2022; Khan *et al*., 2023). This design minimizes the potential for homologous recombination events and contributes to preventing transgene silencing, thereby promoting more robust and sustained expression profiles, which can be utilized for multiple transgenes expression (Kar *et al*., 2022; Yang *et al*., 2023). Hence, progress in enhancing synthetic promoters for genome editing holds the potential to streamline the incorporation of multiple desirable traits (Gehrke *et al*., 2023; Rao *et al*., 2021). Indeed, the recent development of synthetic inducible systems, such as those responsive to light (Ochoa‐Fernandez *et al*., 2020) and copper (Garcia‐Perez *et al*., 2022), in combination with CRISPR‐a tools (Pan and Qi, 2023; Papikian *et al*., 2019; Xiong *et al*., 2021), holds tremendous potential to significantly enhance plant GE and regeneration efficiencies.

Future efforts could usefully focus on integrating synthetic promoter engineering with emerging GE tools, such as native and engineered Cas variants, or CRISPR activation/silencing systems, for fine‐tuned control over spatial and temporal gene expression. Additionally, combining advances in synthetic promoters with inducible systems can provide higher specificity, thereby improving the precision of GE outcomes. Moreover, future research should prioritize scalability and field validation of these synthetic promoter technologies to bridge the gap between lab‐based innovation and real‐world agricultural applications (Altpeter *et al*., 2016; Schaart *et al*., 2016). Ensuring the stability and robustness of synthetic promoters and edited promoters under variable field conditions will be crucial for their widespread adoption in crop breeding programmes and in agriculture more generally (Puchta, 2016; Schindele *et al*., 2020). Strategic investment in interdisciplinary research, involving synthetic biology, bioinformatics and high‐resolution gene regulatory profiling, has the potential to unlock the full potential of synthetic promoters for GE and promoter editing for sustainable agriculture.

Finally, it is worth mentioning that GE technologies have been recently recognized as non‐genetically modified (non‐GM) with or without confirmation by regulatory authorities in the United States, Canada, Russia, India and Australia, while the United Kingdom and China have simplified GMO regulations (Atimango *et al*., 2024; Buchholzer and Frommer, 2023). Thus, by relaxing regulatory constraints on the cultivation and utilization of gene‐edited crop varieties outside the framework of GMO legislation could have a positive influence on the advancement of plant biotechnology and breeding sectors, ultimately contributing to the improvement of sustainable crop development (Buchholzer and Frommer, 2023; Pixley *et al*., 2022). In this context, strengthening GE technologies is crucial for future sustainable development and food security, where promoters, alongside other elements such as engineered polycistronic systems, synthetic gene circuits and advances in plant transformation, will play a key role.

## Conflict of interest

The authors declare no conflicts of interest.

## Author contributions

N.G., L.F.Q. and C.S. contributed to the conceptualization of the work. N.G., L.F.Q., L.L., A.K.S., M.K. and C.S. were responsible for writing the original draft. N.G., L.F.Q., L.L., A.K.S., M.K., G.B., P.McK., M.C. and C.S. handled the review, editing and finalization of the manuscript. Figures and tables were prepared by N.G., L.F.Q., L.L., A.K.S. and M.K. Research supervision and funding were provided by C.S.

## Data Availability

Data sharing is not applicable to this article as no new data were created or analyzed in this study.
